# Application of a decision support system for providing better mental health care: the case of the Basque Country (Spain)

**DOI:** 10.1192/j.eurpsy.2022.1617

**Published:** 2022-09-01

**Authors:** N. Almeda, C. García-Alonso, L. Salvador-Carulla

**Affiliations:** 1 Loyola University, Psychology, Seville, Spain; 2 Loyola University, Quantitative Methods, Seville, Spain; 3 University of Canberra, Faculty Of Health, Canberra, Australia

**Keywords:** Mental health services, Policymaking, Efficiency, mental health care

## Abstract

**Introduction:**

Decision Support Systems (DSS) are appropriate tools for guiding policymaking processes in Mental Health (MH) management, especially where a balanced and integrated care provision is required.

**Objectives:**

To assess the performance of a MH ecosystem for identifying benchmark and target-for-improvement catchment areas according to the Balanced Care model.

**Methods:**

The MH provision, distinguishing inpatient, day and outpatient main types of care, has been assessed in the Mental Health Network of Gipuzkoa (Basque Country, Spain) using a DSS, integrating Data Envelopment Analysis, Monte-Carlo Simulation and Artificial Intelligence. 13 catchment areas, defined by a reference MH centre, are the units (universe) for the analysis. The indicators for MH ecosystem performance were: relative technical efficiency, stability and entropy, for identifying both benchmarking and target-for-improvement areas. The analysis of the differences between the two groups can be used to design organizational interventions.

**Results:**

The Mental Health Network of Gipuzkoa showed high global efficiency scores, but it can be considered statistically unstable (small changes in variable values can have relevant impacts on its performance). For a global performance improvement, it is recommended to reduce admissions and readmissions in inpatient care, increase workforce capacity and utilization of day care services and, finally, increase the availability of outpatient care services.

**Conclusions:**

This research offers a guide for evidence-informed policy-making to improve MH care provision in the main types of care and provide aftercare. The characteristics of the area to be improved are critical to design interventions and assess their potential impact on the MH ecosystem.

**Disclosure:**

No significant relationships.

